# The effect of oral probiotics on CD4 count in patients with HIV infection undergoing treatment with ART who have had an immunological failure

**DOI:** 10.1002/iid3.913

**Published:** 2023-06-14

**Authors:** Masoud Mortezazadeh, Saeed Kalantari, Nooshin Abolghasemi, Mitra Ranjbar, Saeedeh Ebrahimi, Abbas Mofidi, Babak Pezeshkpour, Ensieh Sadat Mansouri, Seyed Zia Tabatabaei, Mehdi Kashani

**Affiliations:** ^1^ Internal Medicine Department, Sina Hospital Tehran University of Medical Sciences Tehran Iran; ^2^ Department of Infectious Disease, Antimicrobial Resistance Research Center, Institute of Immunology and Infectious Diseases Iran University of Medical Sciences Tehran Iran; ^3^ Department of Pharmacology Islamic Azad University Pharmaceutical Sciences Branch –Pharmacy School Tehran Iran; ^4^ Department of Infectious Disease, Firoozgar General Hospital Iran University of Medical Sciences Tehran Iran; ^5^ Department of Infectious Disease Alborz University of Medical Sciences Karaj Iran; ^6^ Department of Infectious Disease, School of Medicine Iran University of Medical Sciences Tehran Iran; ^7^ Department of Infectious Disease, School of Medicine Tehran University of Medical Sciences Tehran Iran

**Keywords:** CD4, HIV, immunologic failure, probiotic, virologic response

## Abstract

**Introduction:**

Probiotics are live microorganisms that, when administered in appropriate colonies, can delay the destruction of the immune system and contribute to the maintenance of immunity in HIV patients. Probiotics play an important role in stimulating natural killer T cells, strengthening the functional gut barrier, and reducing systemic inflammation.

**Methods:**

This study was a randomized double‐blind clinical trial involving 30 patients treated with antiretroviral therapy who had experienced immunological failure despite HIV viral suppression. Patients were divided into two equal groups of 15, group (B) received two probiotic capsules daily with a colony count of 10⁹ CFU per capsule containing seven strains, after 3 months they were examined for CD4^+^ counts by flow cytometry, and after a 1‐month washout period the participants who had received probiotics were switched to placebo, and the participants who had received placebo were given probiotics for 3 months, and they were examined for CD4^+^ counts 7 months after the start of the study.

**Results:**

In the first group (A), administration of the placebo resulted in a decrease in CD4 count in the first 3 months (from 202.21 to 181.79, *p* value < .001), which may be due to the natural history of the disease. After probiotics administration, CD4 count increased significantly (from 181.79 to 243.86, *p* value < .001). Overall, after 7 months of study, there was a significant increase in the mean CD count from 202.21 to 243.86 (*p* value < .001). In the second group (B), the administration of probiotics in the first 3 months of the study resulted in a significant increase in the mean CD4 count (from 126.45 to 175.73, *p* value < .001). Termination of treatment with probiotics resulted in a significant decrease (from 175.73 to 138.9, *p* value < .001) but overall the CD4 count at the end of the study was significantly higher than at baseline (*p* value < .001).

## Graphical abstract

The study investigates the effect of probiotics in patients who have experienced immunological failure despite virological suppression.

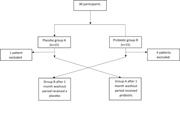

## 1 INTRODUCTION

Antiretroviral therapy (ART) is the basis of treatment for adults with a CD4^+^ count of fewer than 350 cells/μL or with a CD4^+^ count that has any of the following conditions: Concurrent hepatitis B or a sexual partner with this disease, HIV‐induced nephropathy, age > 50, active tuberculosis, a viral load greater than 100,000, or malignancy associated with HIV or malignancy requiring chemotherapy/radiotherapy.^1^, ^2^

Viral load and CD4^+^ count are two factors that determine treatment success.

Virologic failure is defined as an incomplete response or no response from HIV RNA to ART or virologic recurrence, as defined below:

Incomplete virologic response: more than 200 copies of HIV RNA per milliliter after 24 weeks of treatment with HAART or more than 50 copies/mL at Week 48 of treatment in a patient receiving treatment for the first time.^3^

Virologic relapse: HIV RNA up to 400–1000 copies/mL, 4–8 weeks after viral suppression, in two sessions.^3^

Immunologic failure: an increase in CD4 cell count of 150/mm^3^ is expected in the first year of HIV treatment. If this increase is less than 25–50 in the first year, or if the CD4 count does not increase from baseline despite the decreased viral load, or if CD4 cells are consistently below 100 cells/mm^3^ or decrease by 50% of the maximum CD4 count during treatment, immunologic failure is present.^4^, ^5^

Generally, immunologic failure follows virologic failure, and then clinical exacerbations occur. However, these may occur months to years apart and do not necessarily occur in the order mentioned.^4^

In the patients in our study, viral load was minimized and the desired virologic response was achieved, but despite this success, we observed immunologic failure, so according to previous studies we try to increase the gut microflora by probiotics especially with a high count of lactobacilli to reduce intestinal inflammation and achieve an immunologic response.

Probiotics are live microorganisms that, when administered in adequate numbers of colonies, can delay immune destruction and help maintain immunity in HIV patients. A study by Ruben Hummeln et al. investigated the effect of probiotics on the immune system of patients with HIV. The results indicate that at the end of Week 25, the group that received a placebo in combination with ART had an increase of mean of 19 CD4 cells/μL and the group that received probiotics in combination with ART had an increase of mean of 46 CD4 cells/μL.^6^

Several other studies have shown the effect of probiotics on increasing CD4 cell counts. Based on these results, we designed this study to investigate the effect of probiotics in patients who have immunologic failure despite virologic success.

## 2 METHODS

This study was a randomized double‐blind clinical trial involving 30 patients treated with ART at West Medical Center, Tehran, Iran.

According to World Health Organization (WHO) criteria, patients who have received standard first‐line therapy ART for 1 year or more are referred for evaluation of second‐line therapy ART if they have CD4 decline to levels before ART, CD4 decline to less than 50% of peak at baseline, or failure to achieve CD4 levels above 100 c/mm^3^ or those with HIV RNA 10,000 copies/mL or greater qualified for conversion to second‐line therapy ART.

Before the study began the patients received at least 48 weeks of ART (first line or second line) who had experienced immunological failure despite HIV viral suppression.

Patients were divided into two groups of 15. Group (B) received two probiotic capsules daily with a colony count of 10⁹ CFU per capsule containing seven strains (including *Lactobacillus casei*, *Lactobacillus acidophilus*, *Lactobacillus rhamnosus*, *Lactobacillus bulgaricus*, *Bifidobacterium breve*, *Bifidobacterium longum*, *Streptococcus thermophiles* with prebiotic fructooligosaccharides.

By the end of Month 3, patients were tested for CD4^+^ count using flow cytometry and were asked to fill out questionnaires concerning the history of diarrhea or intake of the antibiotic, especially Cotrimoxazole.

After a 1‐month washout period, the participants who had received probiotics were switched to a placebo, and the participants who had received placebo were given probiotics for 3 months, and they were examined for CD4^+^ counts 7 months after the start of the study.

### 2.1 Inclusion criteria

The inclusion criteria included:

1. Patients with a plasma level HIV RNA of less than 200 HIV‐1 RNA copies/mL or a decrease in plasma viral load of more than 1 log10.
2. Patients with immunologic failure if CD4 count decreased to pretherapy baseline (or below) or decreased by 50% from the peak during treatment (if known) or CD4 count remained below 100 cells/mm^3^ 6 months after ART initiation.

### 2.2 Exclusion criteria

The exclusion criteria included:

1. Patients taking antibiotics during the study,
2. Patients with concurrent hepatitis B and C,
3. Patients not adhering to pill regimen,
4. Pregnancy during the study,
5. Active infectious diseases,
6. Surgical procedures 6 months before or during the study on the gastrointestinal tract,
7. Severe liver or kidney failure.

### 2.3 Determination of CD4^+^ count

The sample needed for this test is whole blood collected with EDTA anticoagulant. The measurement of CD4^+^ cells is based on the flow cytometry method. In this method, the blood is first incubated with specific amounts of antibodies conjugated with fluorescent substances such as phycoerythrin, the antimarker to be measured; the cell suspension is then analyzed in a flow cytometer and the values are recorded.

### 2.4 Statistical analysis

The *χ* ^2^ test was used for the comparison of qualitative variables and the *t* test or Mann–Whitney *U* test was used for the analysis of quantitative variables. The effect of probiotics was studied using the logistic regression model.

## 3 RESULT

Thirty patients participated in the study, 5 women and 25 men, and were randomly assigned to two groups. During the first 3 months, five patients voluntarily withdrew from the study (four in the experimental group, and one in the placebo group). We continued the study to the end with 25 patients, 5 women (20%) and 20 men (80%). Our groups included 56 and 44 percent of patients.

The average age of the remaining subjects was 41.4 years (30.8 years for women, 43.9 years for men) and the average duration of treatment with ART was 4.4 years.

The mean CD4 count before the administration of probiotics was 168.88 (SD = 71.072) in all patients. After our first intervention, the administration of placebo and probiotics for groups A and B, we observed a significant increase in CD4 count in group A compared with group B (mean of CD4 count changes: Group A = −20.4286, Group B = 49.27, a difference(A−B) = −69.70, 95% CI: (−97.96 to −41.43), *p* value < .001).

In our second intervention, we exchanged the two groups and administered probiotics for group B and placebo for group A. Again, we observed a significant increase in CD4 counts in the probiotic group compared with the previous phase (mean of CD4 count changes: Group A = 62.071, Group B = −36.81, a difference(A−B) = 98.88, 95% CI: (70.66 to 127.11), *p* value < .001).

Next, we examined the effect of probiotics on each group separately. We wanted to see how the beginning and the end of taking probiotics affected the CD4 count. In group A, administration of the placebo resulted in a decrease in CD4 count in the first 3 months (from 202.21 to 181.79, *p* value < .001), which could be due to the natural history of the disease. After probiotics administration, CD4 count increased significantly (from 181.79 to 243.86, *p* value < .001). Overall, after 6 months of study, there was a significant increase in mean CD count from 202.21 to 243.86 (*p* value < .001).

In group B, the administration of probiotics in the first 3 months of the study resulted in a significant increase in mean CD4 count (from 126.45 to 175.73, *p* value < .001). Termination of treatment with probiotics resulted in a significant decrease (from 175.73 to 138.9, *p* value < .001), but overall the CD4 count at the end of the study was significantly higher than at baseline (*p* value < .001) (Table 1).

**Table 1:** Comparison of mean CD4 changes in groups A and B using the paired *t* test.

Groups	Mean CD4 difference	Standard deviation	*p* Value
Placebo group (A) first 3 months (*n* = 14)	−20.4286	36.35237	.001
Probiotic taken group (B) first 3 months (*n* = 11)	49.2727	30.44697	.001
Probiotic group (A) 2nd 3 months (*n* = 14)	62.0714	40.48070	.001
Placebo group (B) 2nd 3 months (*n* = 11)	−36.8182	22.53361	.001
Group (A) after 3 months (*n* = 14)	41.65	20.825	.001
Group (B) after 3 months (*n* = 11)	12.45	6.225	.001

Our analyses on other variables such as age, gender, duration of treatment with ART, and duration of disease did not reveal significant associations.

## 4 DISCUSSION

The intestinal microbiota plays an essential role in stimulating the development and maintenance of the intestinal immune system.

HIV studies showed that HIV can wreak havoc in the gut, where there are many CD4 cells, This happens fairly soon after infection with HIV.^7^

Gastrointestinal complications are common in people living with HIV, including intestinal barrier impairments, mitochondrial dysfunction, and alterations in the microbiota. These complications are thought to be caused by chronic inflammation, immune activation, and the use of ART.

Intestinal barrier impairment refers to the loss of integrity of the intestinal mucosa, which can lead to increased permeability and transfer of contaminants from the intestine into the bloodstream. This can trigger immune activation and inflammation, which can further damage the intestinal barrier and exacerbate the problem.

Mitochondrial dysfunction is a common complication of PLWH and is associated with consumption of ART. Mitochondria are responsible for energy production in cells, and when they are dysfunctional, cells cannot produce enough energy to function properly. This can lead to a range of symptoms, including fatigue, muscle weakness, and cognitive impairment.^8^

In addition, modern antiretroviral drugs, although well tolerated, cause severe gastrointestinal symptoms such as nausea, vomiting, bloating, or diarrhea in mild to severe forms.^9^

Previous studies demonstrated that Yoghurt enrichment with probiotics as a snack may increase CD4 levels and protect against some HIV‐related gastrointestinal infections and local inflammations.

This local inflammation can lead to new collagen formation and fibrosis, which contribute to the deletion of CD4^+^T cells and limit the recovery of the immune response. An increased predisposition to collagen neogenesis may be related to a decreased population of virgin CD4^+^T lymphocytes whose phenotype is not activated before ART. Fibrosis may be one of the causes of failure of immune recovery during treatment despite suppressed viral replication.^10^

After the HIV virus infects the CD4 cells, it plants itself in several organs, including the MALT system of the digestive system. In this organ, the HIV implants itself in up to 10 times the amount than in the bloodstream.

At this stage, the virus begins to mix its genome with that of the intestinal lymphoid cells. Treatment with ART can combat the virus before this fusion, but after genetic fusion and entry into the silent phase, the virus is inaccessible to the immune system and antivirals.

Probiotics with their effect on enteric translocations bring these infected cells out of their silent phase and expose them to the immune system and pharmacological agents, thereby reducing the burden on the infected reservoir.

In addition to restoring the balance of the gut microbiota in terms of competition with pathogens and improving the intestinal barrier, probiotics also play an important role in restoring mucosal immune function through the Th17/Treg ratio, which reduces systemic and local inflammation in the gut.^11^

Probiotics also may promote epithelial healing by altering intestinal flora and reduce the risk of viral transmission and hospitalization for coinfection by preventing the decline in CD4^+^ cell count. ART‐treated patients who do not show an immunological response (CD4 < 200) have lower lactobacilli levels, increased LPS, and sCD14 levels, and increased inflammatory markers such as IL −6 and sCD14.^12^

So in this study, we administered a kind of probiotics with a high count of lactobacilli then this intervention can lead to reducing inflammation in intestinal mucosa and make a good response for HIV patients with immunologic failure.

Our results show that the administration of probiotics increased CD4 cell counts compared with the placebo. After discontinuation of probiotics, CD4 counts approached baseline values again but were still significantly higher.

Probiotic administration resulted in an average increase of 62 and 49 CD4 cells/μL in both groups, respectively.

our study showed that the number of CD4^+^ cells increases by taking probiotics but decreases back to baseline levels after stopping probiotics. The results could also mean that by administering probiotics for a longer time, we could expose more infected cells and achieve a better result.

On the other hand, previous studies showed that a CD4 count of <50 cells/mm^3^ was a main predictor of treatment failure. This finding may be because patients with baseline CD4 counts of ≤100 cells/mm^3^ have reduced immunity and their response to initial treatment ART may be unsatisfactory.^4^

Therefore longer period probiotic intervention in this group of study can help them to overcome immunologic failure in persistent CD4 counts above 100 cells/mm^3^.

It is also recommended to consider other Reasons cited for immunologic failure including the role of the thymus gland, which declines with age, and drug toxicity of zidovudine, which clinically causes anemia, neutropenia, pancytopenia, and granulocytopenia in 45% of recipients of this drug.^13^

In this type of failure, it is also recommended to first rule out other causes of immunosuppression, such as HIV2, HTLV1, HTLV2, and drug toxicity, and the combination of didanosine and tenofovir is also among the causes that lead to a decrease in CD4 increase and a lack of appropriate immunological response.^14^

In our study, we excluded all the mentioned causes of immunologic failure.

In another study by Livia Trois, conducted on 77 HIV‐infected children aged 2–12 years, they were divided into two groups: one group received probiotics containing *Bifidobacterium bifidum* with *Streptococcus thermophilus* 2.5 × 10^(10)^ CFU for 2 months, and the other group received a standard diet (control group). CD4 counts were collected at baseline and the end of the study. The mean CD4 count increased in the probiotic group (791 cells/mm^3^), whereas it decreased slightly in the control group (538 cells/mm^3^). The change in mean CD4 cell count from baseline was +118 versus −42 cells/mm^3^ in the children receiving the probiotic diet and the control group, respectively (*p* = .049).^15^

In a similar study from Tanzania after the initiation of yogurt‐enriched probiotic consumption, an additional increase of 0.28 cells/μL/day (95% CI: 0.10–0.46, *p* = .003) was observed. After adjustment for the duration of antiretroviral drug use, the additional increase explained by yogurt consumption remained at 0.17 cells/μL/day (95% CI: 0.01–0.34, *p* = .04) which was also significant.^16^

Notwithstanding the aforementioned studies, the pilot clinical trial ACTG 5350 aimed to investigate the potential of targeting the gut microbiota of vertically HIV‐infected children to reduce inflammation and immune activation. In the study, a probiotic preparation supplement containing *Lactobacillus rhamnosus* GG was administered to a group of HIV‐infected children aged 2–10 years. The probiotic preparation was administered for a period of 12 weeks, and the composition of the children's gut microbiota and their immune and inflammatory markers were monitored throughout the study.

No significant differences were observed when comparing the changes in CD4^+^ and CD8^+^ T‐cell counts or CD4/CD8 ratio according to the intervention group, and the differences in CD4 counts between the intervention groups disappeared during the follow‐up period, which contradicted the results of our study.^17^

The small sample size is an important limitation in our study, but we tried to solve this important problem with the cross‐over study design.

the main reason for this limitation was that only 13.59% of all HIV patients have immunological discordance, but this group was the best choice to study the effect of probiotics in HIV patients.^18^

**Figure 1:**
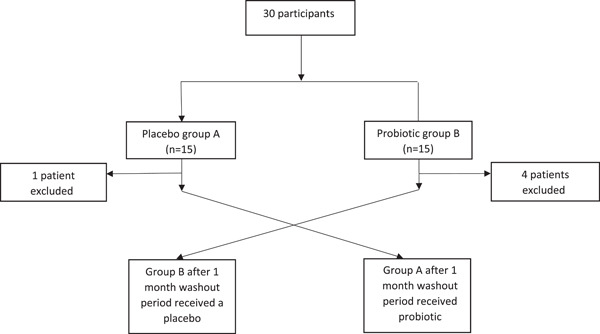
Patients' intervention tree diagram.

Compared with previous studies, the patients we studied suffer from immunologic failure, and given the low CD4 count of the patients in our study, most are classified as stages 3 and 4 of AIDS. This shows the importance of CD4 count, as their low levels can cause numerous clinical adverse events and increasing CD4 count can significantly improve a patient's quality of life (Figure 1).

We also propose to compare the effect of probiotics with new medications such as pidotimod. Pidotimod is an immunomodulatory drug that has been studied for its potential to improve immune function in HIV‐infected individuals and may be able to increase CD4^+^T cell counts and decrease viral load in HIV patients.^19^

## 5 CONCLUSION

the use of probiotics to prevent and attenuate various gastrointestinal manifestations and improve the immunity of intestinal‐associated lymphoid tissue in HIV infection by modulating epithelial barrier functions and microbiota composition can result in a CD4^+^ count rise in HIV patients with immunologic failure

Daily administration of probiotics at appropriate doses‐two 10⁹ CFU capsules in our study‐in HIV patients who had immunologic failure despite virologic response led to a transient increase in CD4^+^ counts.

## AUTHOR CONTRIBUTIONS

**Masoud Mortezazadeh**: Formal analysis; investigation; methodology; supervision; writing—original draft; writing—review and editing. **Saeed Kalantari**: Supervision; writing—original draft; writing—review and editing. **Nooshin Abolghasemi**: Writing—original draft. **Mitra Ranjbar**: Supervision; writing—original draft. **Saeedeh Ebrahimi**: Supervision; writing—original draft. **Abbas Mofidi**: Writing—original draft; writing—review and editing. **Babak Pezeshkpour**: Investigation; writing—original draft. **Ensieh Sadat Mansouri**: Writing—original draft. **Seyed Zia Tabatabaei**: Writing—original draft. **Mehdi Kashani**: Writing—original draft; writing—review and editing.

## CONFLICT OF INTEREST STATEMENT

The authors declare no conflict of interest.

## ETHICS STATEMENT

In this study, no additional costs were imposed on the patients. We maintained the patients' privacy, and their written consent was obtained. The trial was registered with the ethical committee of the Iran University of medical sciences with an ethical number of (IR.IUMS.REC 1395.8821215220). The patients had consented to participation in this article. The participants had consented to the publication of this article.

## Data Availability

The data that support the findings of this study are available from the corresponding author, Masoud Mortezazadeh, upon reasonable request.
